# Filtration Performance Degradation of In‐Use Masks by Vapors from Alcohol‐Based Hand Sanitizers and the Mitigation Solutions

**DOI:** 10.1002/gch2.202100015

**Published:** 2021-06-27

**Authors:** Weidong He, Yinghe Guo, Jingxian Liu, Yang Yue, Jing Wang

**Affiliations:** ^1^ Filter Test Center Northeastern University Shenyang Liaoning CN‐110819 China; ^2^ Institute of Environmental Engineering ETH Zürich Zürich CH‐8093 Switzerland; ^3^ Lab of Advanced Analytical Technologies Empa Dübendorf CH‐8600 Switzerland

**Keywords:** alcohol‐based sanitizers, COVID‐19, filtration efficiency, hand disinfection, masks

## Abstract

In the current COVID‐19 pandemic, wearing masks and hand disinfection are widely adopted hygiene practices. Alcohol‐based sanitizers are commonly used for hand disinfection, however, the alcohol vapors can dissipate the charges on electrostatic filters. In the present study, the effects of alcohol vapors from alcohol‐based sanitizers during hand disinfection on the in‐use masks are studied. The results show that the negative effects are not significant for nonelectrostatic cotton masks or N95 respirators with multiple charged layers, but noticeable for surgical masks. After five rounds of hand disinfection, the filtration efficiencies of the filtering materials of the surgical masks decrease by more than 8% for 400 and 500 nm particles and by 3.7 ± 1.8% for 1 µm particles, the effective filtration efficiency of the surgical masks worn by the volunteers (with leakage considered) decreases by about 5% for ambient aerosol. In another process to imitate intensive disinfection procedures by healthcare workers, a 30 min surface cleaning process using alcohol‐based sanitizer is performed, and the effective efficiency of the N95 respirators worn by the volunteers decreases by nearly 9%. The simple practice of avoiding vapor during hand disinfection could mitigate the effects of alcohol vapor, which is demonstrated on two brands of surgical masks.

## Graphical abstract

Due to COVID‐19, wearing masks and hand disinfection are now widely adopted hygiene practices. Alcohol‐based hand sanitizers can dissipate the charges on electrostatic filters, causing filtration performance degradation for in‐use masks. The tests show that the negative effects are not significant for N95 respirators with multiple charged layers, but noticeable for surgical masks. Simple vapor‐avoiding hand disinfection could mitigate the effects.

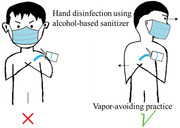

## 1 Introduction

The COVID‐19 pandemic is raging and many countries are suffering the second wave. Compared with the early stage of the pandemic outbreak, more comprehensive approaches, including vaccination, contact tracing, quarantine, physical distancing, hand hygiene, and masks, have been proposed to slow down the spread of COVID‐19.^[^ ^1^ ^]^ Nevertheless, the number of new infections per day has been constantly high since October 2020.^[^ ^2^ ^]^ In order to contain the second wave of the pandemic and keep businesses open, regular hand disinfection and mandatory face masks in public places have been ordered or recommended in the latest anti‐COVID measures by most countries.^[^ ^3^, ^4^, ^5^ ^]^

Since the outbreak of the current pandemic, various studies about masks have been reported. In brief, there are three major mask‐related topics. In the early stage of the pandemic, the effectiveness of masks against the COVID‐19 spreading was discussed.^[^ ^6^, ^7^, ^8^ ^]^ So far, a number of studies showed that wearing masks in public could prevent interhuman transmission effectively.^[^ ^9^, ^10^ ^]^ Second, the effective performance of masks on fitted protection and source control was considered in some studies.^[^ ^11^, ^12^ ^]^ Third, a number of studies about mask regeneration and alternative materials have been performed to address the mask shortage issue.^[^ ^13^, ^14^, ^15^, ^16^, ^17^ ^]^ The reliability of masks when they are exposed to high temperature, high humidity, and chemical agents during regeneration process is well understood.^[^ ^12^, ^17^, ^18^, ^19^ ^]^ However, more studies are still needed to understand the reliability of in‐use masks. Most surgical and personal protective masks are made of polypropylene (PP) electrostatic materials which provide enough filtration efficiency and low respiratory resistance.^[^ ^20^ ^]^ According to previous studies, the normal environmental conditions and the temperature and humidity from human breath should not impact the filtration performance of in‐use masks.^[^ ^21^, ^22^ ^]^ However, special conditions may need to be considered. For example, it is confirmed that alcohol‐based sanitizers can inactivate the SARS‐CoV‐2 virus, and they are recommended by the World Health Organization (WHO).^[^ ^23^, ^24^ ^]^ Regular hand disinfection and wearing masks in public places will be a necessary part of our life in the foreseeable future. Many hand sanitizers on the market are alcohol‐based. However, organic solvents including alcohol‐based agents can dissipate the charges on electrostatic filters.^[^ ^25^, ^26^ ^]^ Both N95 respirators and surgical masks comprise electrostatic filter medium, therefore the potential risk of applying alcohol‐based sanitizers while wearing masks should be addressed. Although the alcohol‐based sanitizers would not directly contact the masks worn by the users during the hand disinfection, the vapors of alcohol‐based sanitizers could dissipate the electrostatic charges on the masks, finally leading to diminished protection for the mask wearers.

In the present work, the effects of hand disinfection using alcohol‐based sanitizers on filtration performances of cotton masks, surgical masks, and N95 respirators worn by the users were investigated. The dependence of the degradation effect on the number of performed hand disinfection was analyzed. By a process imitating intensive cleaning and disinfection procedures by healthcare workers, the influence of continuous (30 min) alcohol vapor exposure on N95 respirators was studied. In addition, we proposed a simple practice for vapor‐avoiding hand disinfection to mitigate the effects of alcohol‐based sanitizers on mask filtration performance.

## 2 Experimental Section

### 2.1 Materials

A type of N95 respirator, two brands of surgical masks, and a type of cotton mask on the Swiss market were selected to be evaluated, the selected masks were shown in Figure S1 in the Supporting Information. In the present study, the effective filtration areas of surgical masks, N95 respirator, and cotton mask were 210, 173, and 105 cm^2^, respectively. The used alcohol‐based hand sanitizer was 60–80% 2‐Propanol which was one of WHO‐recommended handrub formulations.^[^ ^24^ ^]^

### 2.2 Surface Potential Test

The whole mask was placed on a grounded metal platform, and the surface potential was measured by an electrostatic voltmeter (Monroe 244A). The probe of the electrostatic voltmeter was set at 5 mm above the test sample (Figure S2, Supporting Information). The surface potential of five positions on the sample were measured, and the measurement was repeated for three different pieces.

### 2.3 Filtration Performance Test for Mask Filtering Materials and Whole Masks

**Figure 1:**
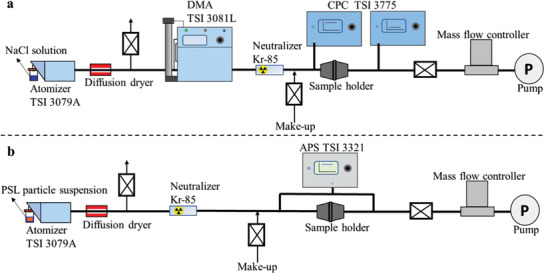
a) Setup for the NaCl particle filtration test in the size range of 50–500 nm; b) Setup for the PSL particle filtration test in the size range of 1–3 µm.

The filtration efficiencies of the mask filtering materials and the in‐use whole masks were measured separately. For the filtration test of mask filtering material, a circular sample with a diameter of 4.5 cm was cut from the mask, then the filtration efficiency of the circular mask sample for 50–500 nm particles were measured to evaluate the filtration performance changes (**Figure** **1**a). Briefly, an atomizer was used to generate polydisperse particles, and a differential mobility analyzer (DMA, TSI 3081L) was employed to select monodisperse particles. The selected monodisperse particles were neutralized by a radioactive source (Kr‐85). Two condensation particle counters (CPC, TSI 3775) were used to measure the particle concentrations of upstream and downstream samples. A sheath to aerosol flow ratio (SAFR) of 10 in the DMA was applied in order to limit the artifact due to the multiply charged particles. For the filtration efficiency test of 1 and 3 µm particles, the monodisperse polystyrene latex (PSL) particles were employed. An aerodynamic particle sizer (APS, TSI 3321) was used to measure the particle concentrations up‐stream and down‐stream (Figure 1b). A commonly used test velocity of 5.3 cm s^−1^ for fabric filter and personal protection devices was applied to test the circular mask samples.^[^ ^27^ ^]^ The test velocity corresponded to breathing rates of 33, 55, and 67 L min^−1^ for the studied surgical mask, N95 respirator, and cotton mask, respectively. More details of the particle filtration test can be found in the previous study.^[^ ^13^ ^]^ The particle sizes were selected according to the report that aerosols containing SARS‐CoV‐2 were found in the size range of 250–1000 nm, and the standard test (EN 14 683) for surgical masks was at 3 µm particles.^[^ ^28^ ^]^ The size distributions of monodisperse particles used for the filtration test are shown in Figure S3 in the Supporting Information.

The test setup (Figure S4, Supporting Information) for the effective filtration efficiency (with leakage considered) of the whole masks worn by volunteers followed the Ambient Aerosol Condensation Nuclei Counter (CNC) Quantitative Fit Test (QNFT) Protocols in 29 CFR 1910.134, Appendix A.^[^ ^29^ ^]^ A CPC was employed to measure the concentrations of the outside particles and the particles inside the in‐use mask via a sampling tube. The tube was placed inside the mask through a hole on the mask, the gap between the tube and the hole was sealed with silicone. The temperature and relative humidity of ambient air during the test was 25 °C and 40–60%, respectively. The size distribution of ambient particles is shown in Figure S5 in the Supporting Information. During the test, the volunteers breathed normally and spoke occasionally.

To imitate intensive disinfection procedures by healthcare workers, five volunteers wearing N95 respirators cleaned lab tables, floor, and instruments by using alcohol‐based hand sanitizer spray and paper towel wetted by the sanitizer. After 30 min, the effective filtration efficiency of N95 respirators for ambient aerosol was measured via the QNFT protocols.

## 3 Results and Discussion

### 3.1 Two Types of Hand Disinfection Practices Featuring Different Hand and Face Positions

**Figure 2:**
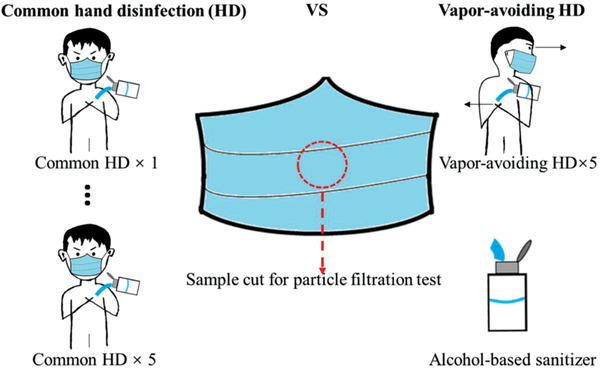
Using alcohol‐based sanitizer for common and vapor‐avoiding hand disinfection.

WHO published a guide for the detailed hand disinfection steps, but the position of hands during hand rubbing was not mentioned.^[^ ^30^ ^]^ Herein, the WHO recommended hand disinfection steps were employed. The duration of the entire procedure was 20–30 s. In one type of practice illustrated on the left of **Figure** **2**, the volunteers placed hands between the abdomen and chest, which was named as the common hand disinfection in the present study. In the other type of practice, to avoid inhaling the sanitizer vapor, the volunteers placed hands on one side of the body and turned the head to the opposite side, as shown on the right in Figure 2. The second practice was named as the vapor‐avoiding hand disinfection. For comparison, the performances of brand new masks and masks worn by the volunteers for 5 h were tested. Common hand disinfection and vapor‐avoiding hand disinfection using 60–80% 2‐Propanol were performed up to 10 times, then the part near the nose/mouth area of the mask where the inhaled airflow was most concentrated was cut out for the performance test. In the figures, “No HD” indicates the mask was worn 5 h without hand disinfection; “HD × *n*” indicates that *n* times of hand disinfection were performed during the 5 h when the mask was worn.

### 3.2 The Influence of Alcohol Vapors on the Filtering Material of a Cotton Mask

**Figure 3:**
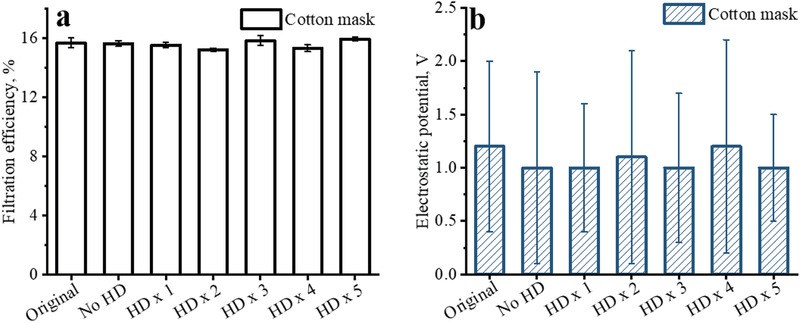
Filtration efficiencies and surface potentials of the filtering materials of brand new and used cotton masks without and with several times of common hand disinfection (HD); a) Filtration efficiency; b) Surface potential.

Because the filtration efficiencies of the selected cotton mask for 50–800 nm particles were very low (about 10–20%, Figure S6, Supporting Information), we only used the total filtration efficiency of the cotton mask for the polydisperse NaCl particles (Figure S7, Supporting Information) to evaluate the effect of exposure to sanitizer vapor. The cotton mask consisted of textile fabric, and its particle capture function only depended on the physical structure instead of electrostatic property. Both the filtration efficiencies and surface potential of the cotton mask had no change after 5 times of common hand disinfection (**Figure** **3**; Figure S8, Supporting Information).

### 3.3 The Surface Potential and Filtration Efficiency of the Filtering Materials of N95 Respirators After 10 Times of Common Hand Disinfection

**Figure 4:**
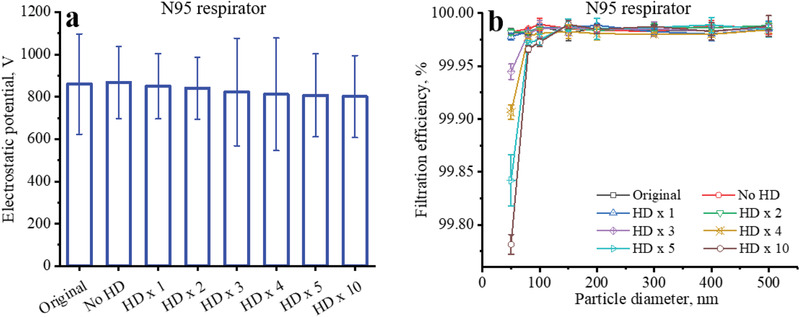
Surface potentials and filtration efficiencies of the filtering materials of N95 respirators a) without and b) with several times of common hand disinfection (HD).

The surface potential of all tested N95 respirators had no statistically significant difference after common hand disinfection up to 10 times, which indicated that using alcohol‐based sanitizers would not influence the electrostatic property of N95 respirators under such experimental conditions (**Figure** **4**a). Common hand disinfection up to 10 times had no obvious influence on the filtration efficiencies of the N95 respirator for particles in the range of 80–500 nm (Figure 4b). After 10 times of common hand disinfection, the filtration efficiency of the N95 respirator for 50 nm particles decreased slightly from 99.98 ± 0.003% to 99.78 ± 0.01%, which could still provide high level protection.

### 3.4 The Filtration Efficiency Changes of the Filtering Materials of Surgical Masks with the Increasing Number of Common Hand Disinfection

**Figure 5:**
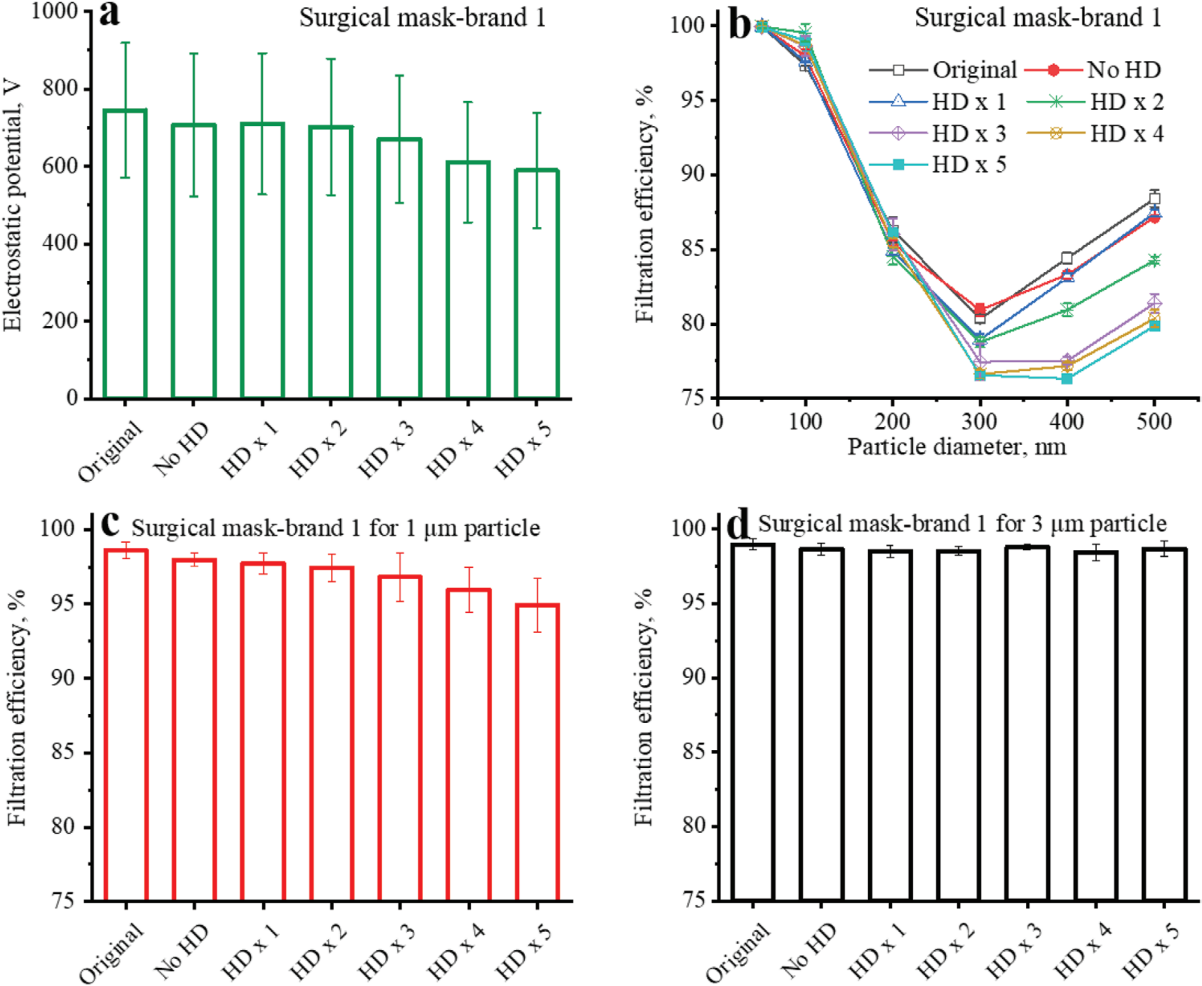
Surface potentials and filtration efficiencies of the filtering materials of brand new and used surgical masks (brand 1) without and with several times of common hand disinfection (HD).

For surgical masks (brand 1), the average electrostatic potential decreased as the number of common hand disinfection increased (**Figure** **5**a). A statistically significant degradation of the surface potential occurred when the number of hand disinfection increased to 4 times or more (Table S1, Supporting Information). As shown in Figure 5b, the filtration efficiencies of the surgical masks (brand 1) had almost no change after 5 h usage without hand disinfection. In comparison, 1.4 ± 0.4% degradation of the filtration efficiency for 300 nm particles was observed for the surgical masks with 1 time of common hand disinfection. After 2 times of common hand disinfection, the filtration efficiencies of the surgical masks for 400 and 500 nm particles decreased from 84.4 ± 0.3% and 88.4 ± 0.5% to 80.9 ± 0.4% and 84.2 ± 0.2%, respectively. Consistent with the drop in the surface potential, the filtration efficiencies of the surgical masks decreased as the number of common hand disinfection increased (Figure 5b). After 5 times of common hand disinfection, the degradation of filtration efficiency for 300 nm particles exceeded 3%, and more than 8% filtration efficiency degradation for 400 and 500 nm particles was observed. After 4 and 5 times of common hand disinfection, the filtration efficiency for 1 µm particles decreased from 98.6 ± 0.5% to 95.9 ± 1.4% and 94.9 ± 1.8%, respectively (Figure 5c). The filtration efficiency for 3 µm particles was not affected (Figure 5d). The different alcohol vapor effects for various particle sizes were attributed to the underlying filtration mechanisms: electrostatic capture plays a significant role for small particles in the sub‐micrometer range, whereas interception and inertial impaction dominate for particles above several micrometers.^[^ ^31^ ^]^ The aerosols containing SARS‐CoV‐2 were found in the size range of 250–1000 nm.^[^ ^28^ ^]^ Therefore, the filtration efficiency degradation of surgical masks after common hand disinfection for several times would diminish the protection for the mask wearers who are exposed to airborne SARS‐CoV‐2 aerosols.

The charge de‐trapping of electrostatic filters induced by alcohol vapor was the main reason of the filtration efficiency degradation, which was shown in our previous study.^[^ ^25^ ^]^ Actually, alcohol vapor treatment is a standard method in ISO/DIS 16890–1:2016 to discharge electret filters and has been widely used in previous studies.^[^ ^32^, ^33^ ^]^ It has been noticed that the same common hand disinfection exhibited different influences on surgical masks and N95 respirators, which might be attributed to the different structures of these two types of masks (Figure S9, Supporting Information). The particle capture function of both the surgical mask and N95 respirator depends on the inner layer which usually consists of charged PP melt‐blown nonwoven. First, the outermost layer of the N95 respirator was thicker than that of the surgical mask. It is more difficult for the alcohol vapor to penetrate into the inner layer of the N95 respirator. Second, the surgical mask had a single charged PP melt‐blown nonwoven inner layer, whereas the N95 respirator possessed multiple nonwoven layers. Although the original surface potentials of the two types of masks were similar, the charge amount throughout the entire N95 respirator was higher than the surgical mask. In other words, the alcohol vapor dose from hand disinfection in the present study only dissipated a small percentage of the charges on the N95 respirator, and was not enough to induce notable degradation of the surface potential or filtration efficiency.

### 3.5 The Effective Filtration Efficiency of Whole Surgical Masks (Brand 2) After 5 Times of Common Hand Disinfection

**Figure 6:**
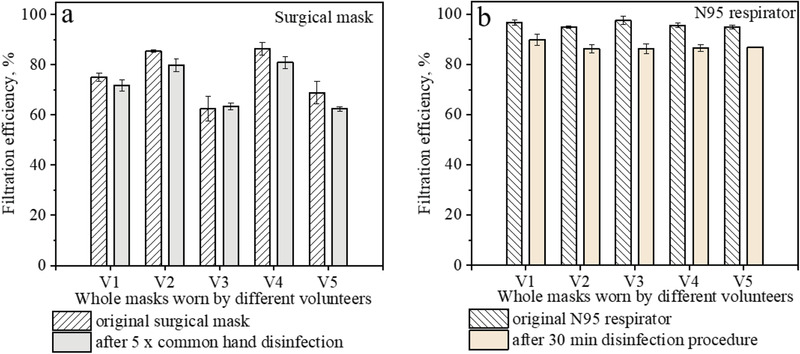
The filtration efficiencies of the masks worn by volunteers without and with several times of common HD; the test particles were ambient aerosol; a) Surgical mask‐brand 2, b) N95 respirator.

The leakage around a mask worn by the user may lead to lower effective filtration efficiency compared to the tested filtration efficiency using the cut‐out filter material. In order to understand the practical effect of alcohol vapors on the in‐use masks, the effective filtration efficiencies of the whole masks worn by five volunteers were evaluated. The test was performed by measuring the total particle concentrations inside and outside of the mask worn by the volunteer exposed to normal ambient aerosol (see details in the Experimental Section). In each test set, three masks worn by the same volunteer were tested to calculate the average filtration efficiency. As shown in **Figure** **6**a, a notable degradation of the effective filtration efficiency of the surgical mask was observed in four out of five test sets after 5 times of common hand disinfection, with the average dropped effective filtration efficiency of 5%.

### 3.6 The Filtration Efficiency of Whole N95 Respirators After Exposure to Alcohol Vapors for 30 min

The total duration of the masks exposed to alcohol vapors was ≈150 s during the five times of hand disinfection, which was not enough to induce notable degradation of the filtration efficiency of the N95 respirator. However, currently N95 respirators are mainly used by medical staff in healthcare settings, where the usage of alcohol‐based sanitizers may be intensive. A previous study revealed that workers from many occupations at hospitals were exposed to various chemicals for long durations, because they spent on average 108–177 min per shift to perform cleaning and disinfecting tasks.^[^ ^34^ ^]^ Alcohol based sanitizers are one of the most common disinfection products, therefore some healthcare workers may be exposed to alcohol vapors for long durations. Herein, we tested the influence of continuous exposure to alcohol vapor on the filtration efficiencies of N95 respirators worn by five volunteers who carried out surface disinfection procedures for 30 min (see details in the Experimental Section). As shown in Figure 6b, the filtration efficiency of the N95 respirators worn by volunteers decreased from 95.9 ± 1.0% to 87.2 ± 1.5% after exposure to the alcohol vapor for 30 min. Such degradation in filtration efficiency of N95 respirators may increase the infection risk when the wearers are exposed to virus‐laden aerosols. Using alcohol free sanitizers would be an efficient method to avert the negative effects on N95 respirators, however, the availability of such products poses limitation and their effects on respirators also need investigation.

### 3.7 Applying Vapor‐Avoiding Hand Disinfection to Mitigate the Negative Influence of Alcohol Vapors on In‐Use Surgical Mask

**Figure 7:**
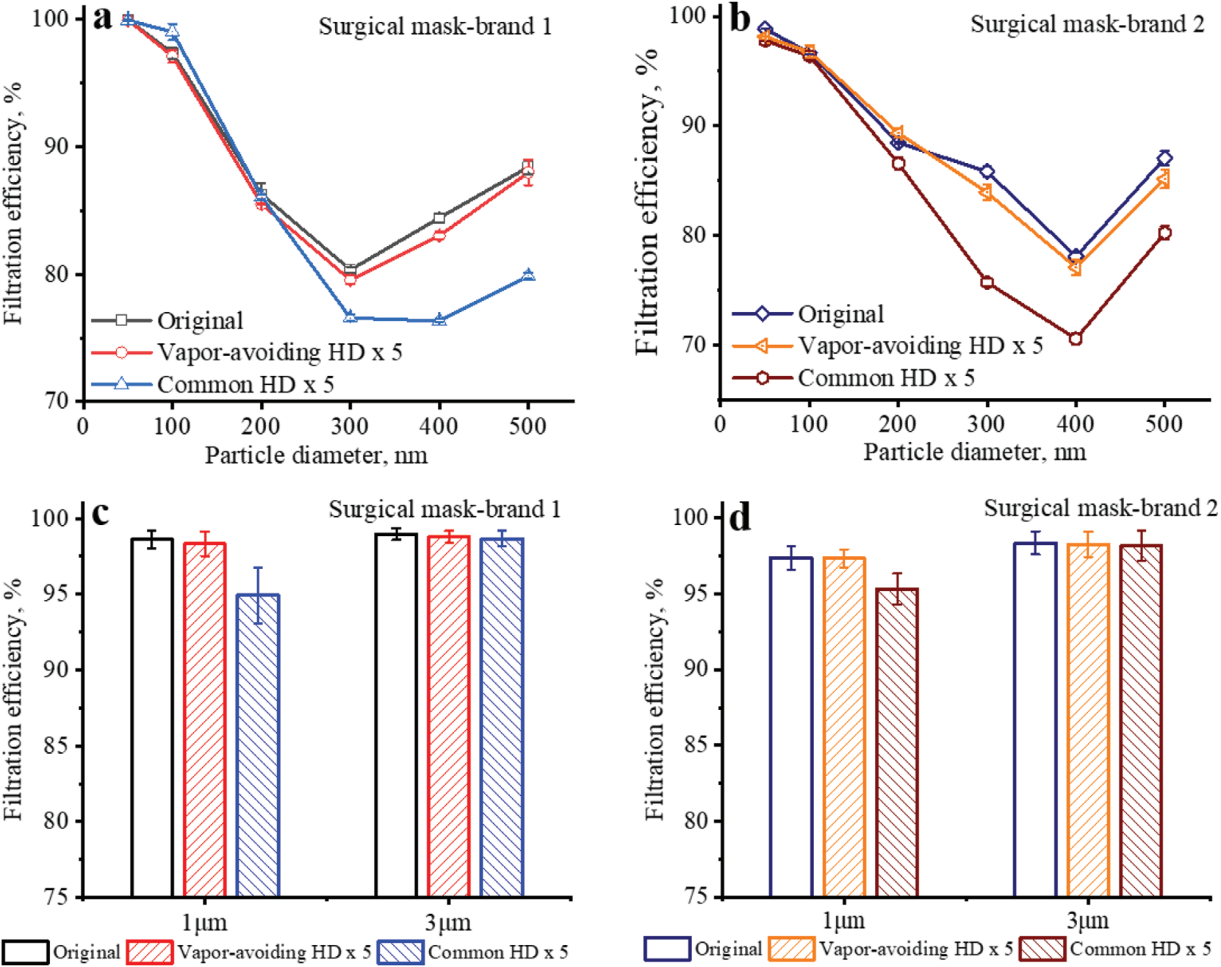
Comparison of the filtration efficiencies of the filtering materials from original surgical masks and surgical masks after 5 times of common hand disinfection and vapor‐avoiding hand disinfection; a,c) Surgical mask‐brand 1; b,d) Surgical mask‐brand 2.

Surgical masks are widely used by general public now, the vapors from alcohol‐based sanitizers during hand disinfection presented more influence on surgical masks than on N95 respirators, thus appropriate mitigation strategies are needed. Herein, we proposed a vapor‐avoiding strategy to mitigate the surgical mask efficiency degradation induced by hand disinfection using alcohol‐based sanitizers. Two brands of surgical mask were tested to evaluate the vapor‐avoiding hand disinfection method. For the filtering material of surgical masks (brand 1), only ≈1% degradation of filtration efficiency for 400 nm particles was observed after 5 times of vapor‐avoiding hand disinfection (**Figure** **7**a). The filtration efficiency for 1 µm particles was also maintained when applying vapor‐avoiding hand disinfection (Figure 7c). In comparison, the degradation of filtration efficiencies for both 400 and 500 nm particles exceeded 8% after 5 times of common hand disinfection. Similar results were obtained for the filtering material of surgical masks‐brand 2 (Figure 7b,d). Other methods such as putting hands behind the body can also provide adequate protection of the mask during hand disinfection using alcohol‐based sanitizers. The key point is to avoid inhaling alcohol vapor.

## 4 Conclusions

In summary, using alcohol‐based sanitizers for hand disinfection may degrade the filtration efficiencies of the in‐use masks, thereby weaken the protection for the mask wearers when they are exposed to virus‐laden aerosols. The negative effects of alcohol vapors from hand sanitizer on the cotton mask and N95 respirator are not significant. Cotton masks are not electrostatic filters and their filtration performance is not influenced by alcohol vapor. However, cotton masks may have low efficiencies and are not commonly used by medical personnel. The strong resistance of the N95 respirator to alcohol vapor was attributed to its thick outermost layer and multiple charged inner layers. Currently N95 respirators are mainly used by medical staff in healthcare settings, where high dose of alcohol‐based sanitizers may be used not only for hand disinfection but also for medical device disinfection. The effective filtration efficiency of N95 respirators worn by volunteers decreased notably when the volunteers performed cleaning and disinfection procedures using alcohol sanitizers for 30 min. By applying the precautionary principle in the case of highly dangerous viruses, the influence of alcohol vapor generated during disinfection processes on N95 respirators should be considered, especially in healthcare settings. For the filtering materials of surgical masks, the degradation of the filtration efficiency for 300 nm particles was observed after one time of common hand disinfection. When the number of common hand disinfection increased to five, the filtration efficiencies of the mask filtering material for 400 and 500 nm particles degraded by more than 8%, and the effective filtration efficiency of the worn whole masks for ambient aerosol decreased by about 5%.

The common hand disinfection used in the present study followed the standard hand disinfection steps recommended by WHO, and the position of the hands was intentionally kept consistent. The individual differences in the hand disinfection steps and body position may cause different effects on the mask performance than those shown here.

There are no shortcuts and only a comprehensive approach can slow down the spread of the current pandemic. Wearing masks is a critical part of the comprehensive prevention measures, therefore more attention should be paid to the reliability of masks. Using alcohol‐based sanitizers for hand disinfection may degrade the filtration performance of masks by dissipating the charges. Vapor‐avoiding hand disinfection is a simple and efficient practice to mitigate such risks. We recommend adding the vapor‐avoiding hand disinfection in the guide of hand hygiene.

## Conflict of Interest

The authors declare no conflict of interest.

## Author Contributions

W.H. and Y.G. contributed equally to this work. W.H., Y.G., and J.W. conceived the research ideas and wrote the manuscript. W.H. and Y.G. conducted the experiments. J.L. and Y.Y. contributed to the data analysis. All authors have discussed the results and have given approval to the final version of the manuscripts.

## Supporting information

Supporting Information

## Data Availability

Research data are not shared.
