# Supplementary material for: Filtration Performance Degradation of In‐Use Masks by Vapors from Alcohol‐Based Hand Sanitizers and the Mitigation Solutions
Source: Glob Chall. 2021 Jun 27;5(9):2100015. doi: 10.1002/gch2.202100015 (PMC8414512; doi:10.1002/gch2.202100015)
Supplement: Supplementary file 1 — Supporting Information [file GCH2-5-2100015-s001.pdf]

© 2021 Wiley-VCH GmbH

Global Challenges

Supporting Information

for *Global Challenges,* DOI: 10.1002/gch2.202100015

Filtration Performance Degradation of In-Use Masks by Vapors from Alcohol-Based Hand Sanitizers and the Mitigation Solutions

Weidong He, Yinghe Guo, Jingxian Liu, Yang Yue, and Jing Wang**

Supporting Information

Filtration Performance Degradation of In-Use Masks by Vapors from Alcohol-Based Hand Sanitizers and the Mitigation Solutions

Weidong He[1,2,3,‡], Yinghe Guo[1,2,3,‡], Jingxian Liu[1,*], Yang Yue[2,3], Jing Wang[2,3,*]

[1] Filter Test Center, Northeastern University, Shenyang, Liaoning, CN-110819, China

[2] Institute of Environmental Engineering, ETH Zürich, CH-8093, Switzerland

[3] Lab of advanced analytical technologies, Empa, Dübendorf, CH-8600, Switzerland

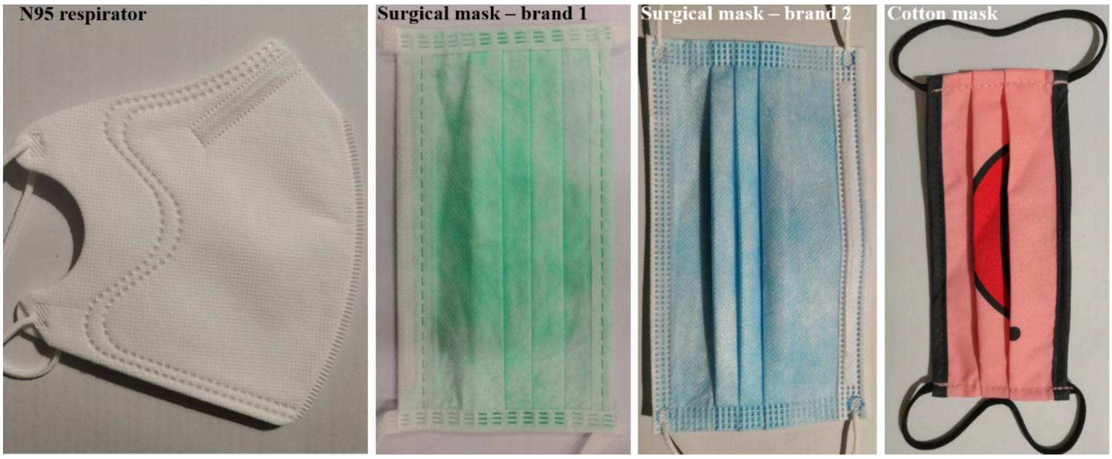

Fig. S1 The N95 respirator, surgical masks (brand 1 and brand 2), and cotton mask used in the present study.

Electrostatic Potential Test. The circular mask samples were put on a grounded metal platform, and the electrostatic potential was measured using an electrostatic voltmeter (Fig. S2). The probe of the electrostatic voltmeter was set at approximately 5 mm above the test sample. The electrostatic potential of the five positions on the sample were measured, and the measurement was repeated for three different pieces.

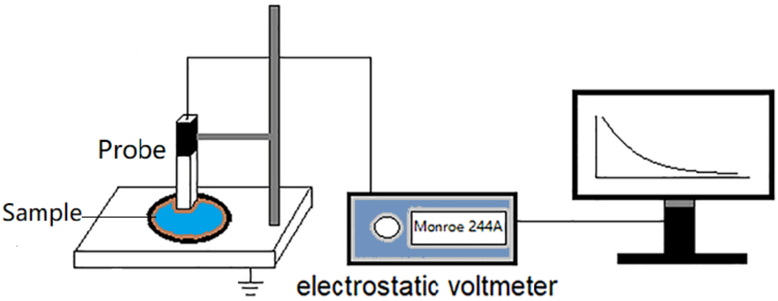

Fig. S2 Schematic of the electrostatic potential test apparatus

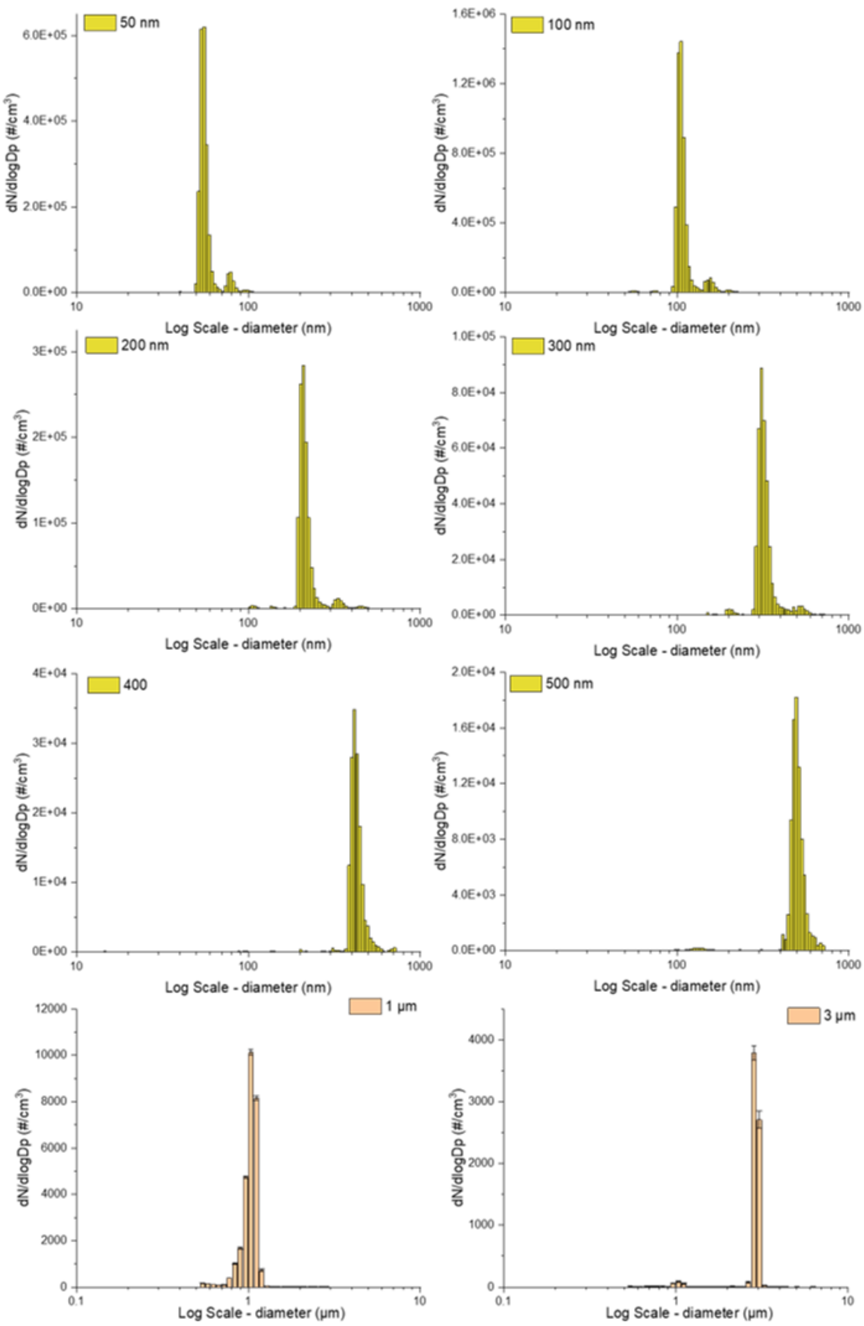

Fig. S3 Size distributions of classified mono-disperse NaCl particles (50-500 nm) and PSL particles (1 and 3 μm) used for the particle filtration test.

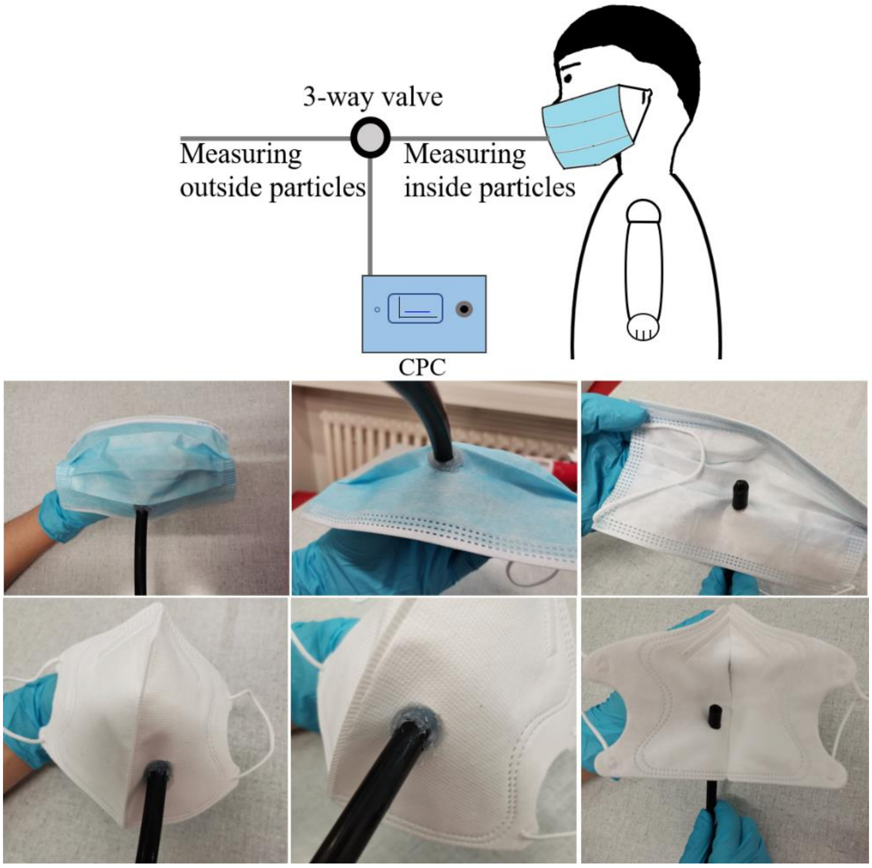

Fig. S4 The measurement of the effective filtration efficiency of masks.

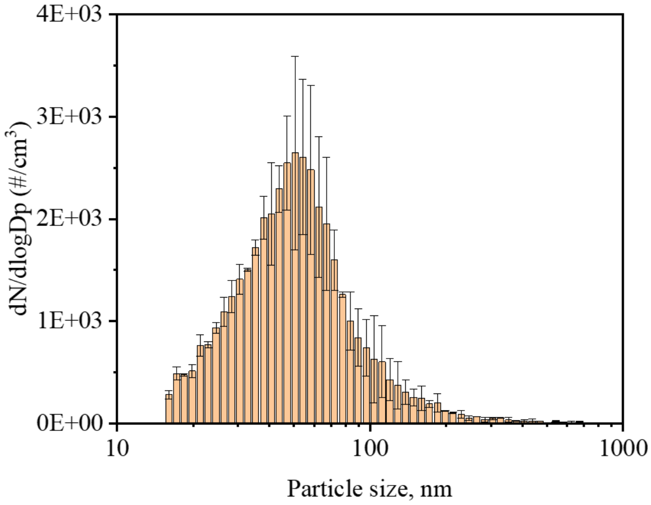

Fig. S5 Size distributions of ambient aerosol in lab condition.

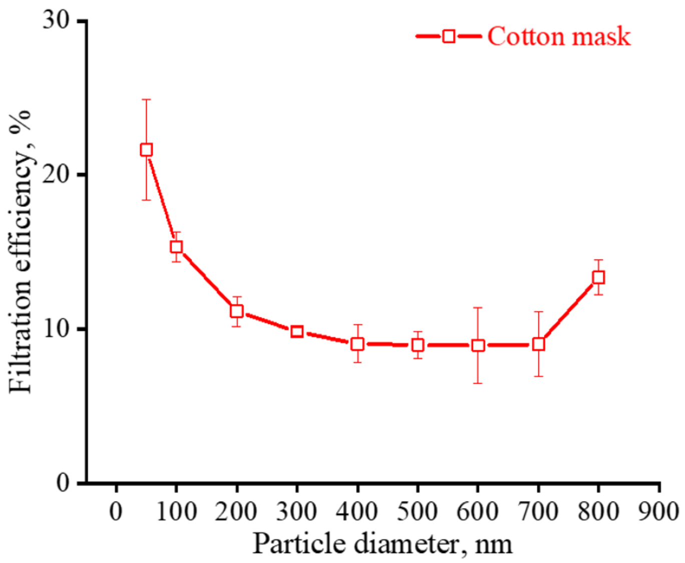

Fig. S6 Filtration efficiencies of the cotton mask for the particles in the range of 50-800 nm.

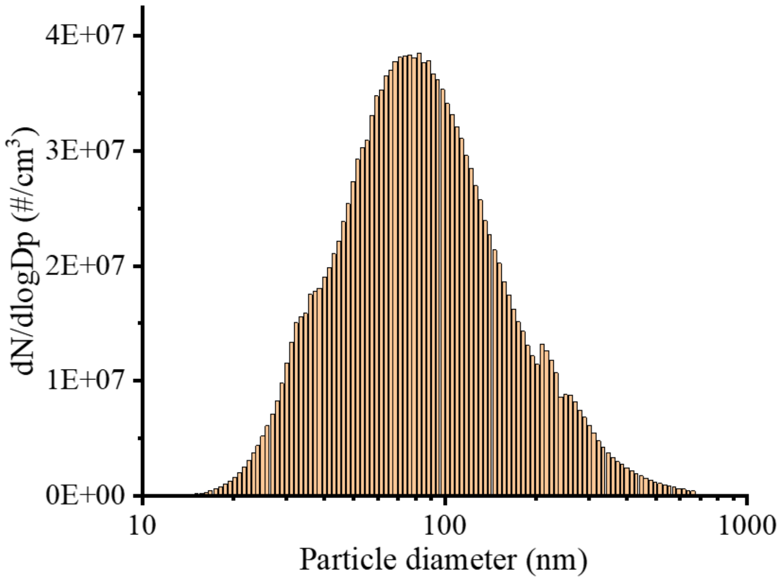

Fig. S7 Size distribution of NaCl particles used for testing the total filtration efficiency of the cotton mask.

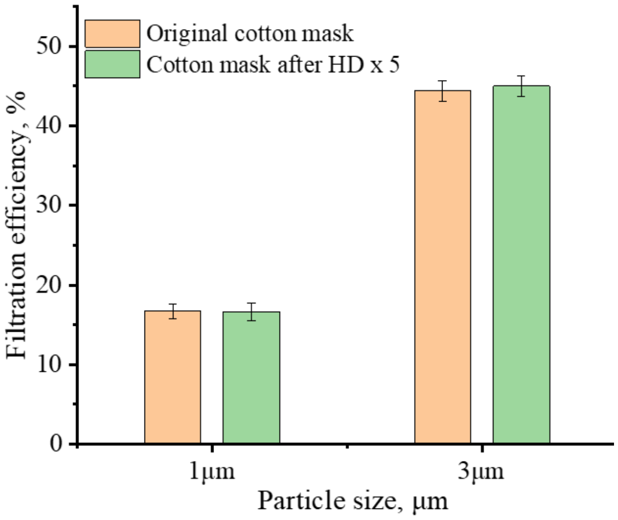

Fig. S8 Filtration efficiencies of brand new and used cotton masks for 1 and 3 μm particles without and with several times of common hand disinfection.

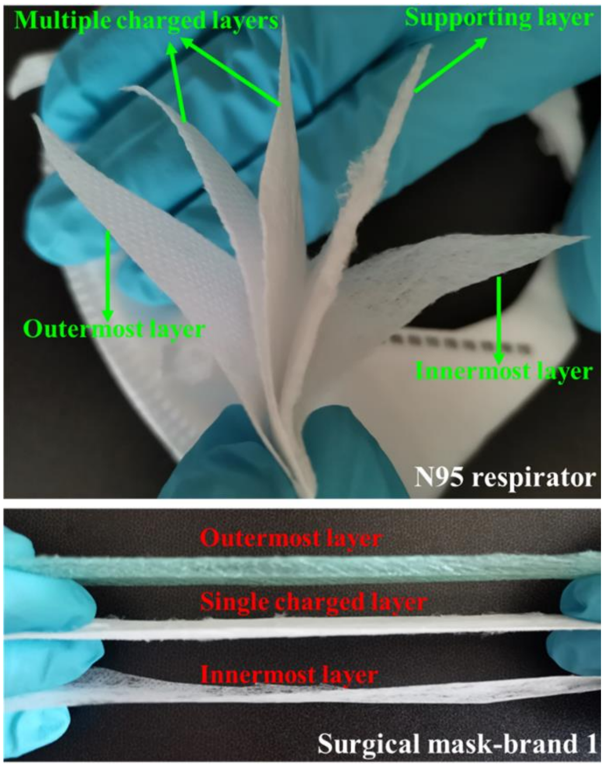

Fig. S9 The structure of multiple layers of the surgical mask and N95 respirator used in the present study.

Statistical Analysis. The data of surgical masks were analyzed using Origin 2018 software. Student's *t-test* was used for the electrostatic potential results analysis. A p-value of less than 0.05 indicated a statistically significant difference compared with

original surgical mask at a confidence level of 95%. The results are shown in Table S1.

Table. S1 The average electrostatic potential (AVG) of surgical masks (brand 1) after different times of common hand disinfection and the statistical analysis in comparison with the original mask.

	Original	No HD	HD x 1	HD x 2	HD x 3	HD x 4	HD x 5
AVG (V)	744	707	710	702	669	610	589
Uncertainty	174	184	182	175	163	154	149
P-value	Na	0.28	0.29	0.25	0.11	0.02	0.007
